# Exploratory Network Meta Regression Analysis of Stroke Prevention in Atrial Fibrillation Fails to Identify Any Interactions with Treatment Effect

**DOI:** 10.1371/journal.pone.0161864

**Published:** 2016-08-25

**Authors:** Sarah Batson, Alex Sutton, Keith Abrams

**Affiliations:** 1 DRG Abacus, 6 Talisman Business Centre, Talisman Road, Bicester, United Kingdom, OX26 6HR; 2 Department of Health Sciences, University of Leicester, Centre for Medicine, University Road, Leicester, United Kingdom, LE1 7RH; Massachusetts General Hospital, UNITED STATES

## Abstract

**Background:**

Patients with atrial fibrillation are at a greater risk of stroke and therefore the main goal for treatment of patients with atrial fibrillation is to prevent stroke from occurring. There are a number of different stroke prevention treatments available to include warfarin and novel oral anticoagulants. Previous network meta-analyses of novel oral anticoagulants for stroke prevention in atrial fibrillation acknowledge the limitation of heterogeneity across the included trials but have not explored the impact of potentially important treatment modifying covariates.

**Objectives:**

To explore potentially important treatment modifying covariates using network meta-regression analyses for stroke prevention in atrial fibrillation.

**Methods:**

We performed a network meta-analysis for the outcome of ischaemic stroke and conducted an exploratory regression analysis considering potentially important treatment modifying covariates. These covariates included the proportion of patients with a previous stroke, proportion of males, mean age, the duration of study follow-up and the patients underlying risk of ischaemic stroke.

**Results:**

None of the covariates explored impacted relative treatment effects relative to placebo. Notably, the exploration of ‘study follow-up’ as a covariate supported the assumption that difference in trial durations is unimportant in this indication despite the variation across trials in the network.

**Conclusion:**

This study is limited by the quantity of data available. Further investigation is warranted, and, as justifying further trials may be difficult, it would be desirable to obtain individual patient level data (IPD) to facilitate an effort to relate treatment effects to IPD covariates in order to investigate heterogeneity. Observational data could also be examined to establish if there are potential trends elsewhere. The approach and methods presented have potentially wide applications within any indication as to highlight the potential benefit of extending decision problems to include additional comparators outside of those of primary interest to allow for the exploration of heterogeneity.

## Introduction

### Atrial fibrillation

Atrial fibrillation (AF) is the most common chronic cardiac arrhythmia and patients with AF are at a greater risk of stroke [1]. Those which experience a stroke are at higher risk of mortality and morbidity [1, 2]. The main goal for treating patients with AF is to prevent stroke from occurring [2]. There are a number of different stroke prevention treatments available but the mainstay treatment for AF is the adjusted dose vitamin K antagonist (VKA) warfarin. However, a number of limitations are associated with the use of warfarin including its associations with bleeding complications, known food and drug interactions, and an adjusted dose mode of administration which requires coagulation dose monitoring [3, 4]. As a result of these limitations, warfarin is associated with high rates of discontinuation in practice [5]. The requirement for alternative anticoagulant agents which are effective, that have favourable safety profiles and are convenient to use has led to the development of novel oral anticoagulants (NOACs) which provide the practical benefits of a fixed dose administration while providing predictable anticoagulation. NOACs are classed as either direct thrombin inhibitors such as dabigatran or direct factor Xa inhibitors such as apixaban, rivaroxaban and edoxaban. The benefits of NOACs compared with warfarin for the prevention of stroke in patients with non-valvular AF has been demonstrated in four large Phase III randomised controlled trials (RCTs); ARISTOTLE [6], ROCKET-AF [7], RE-LY [8] and ENGAGE AF-TIMI 48 [9]. In all of these pivotal studies NOACs demonstrated non-inferior efficacy compared with warfarin for preventing strokes, with similar or more favourable bleeding profiles. Decision makers need to assess the comparative efficacy and safety of NOACs and other anticoagulants for stroke prevention in AF. Network meta-analysis (NMA) offers the most feasible approach to provide this information in the absence of a head to head RCT of all comparators of interest. NMAs should provide a reflection of the evidence base and include reasonable and justified assumptions to provide the basis for decision making.

### Previous NMAs in atrial fibrillation

There are a number of published NMAs in AF, and since the publication of the most recent NOAC Phase III trial for edoxaban [9], five additional NMAs [10–14] have included data from the ENGAGE AF-TIMI 48 trial [9]. Four of these publications restricted evidence networks to include NOAC trials and a single NMA publication included additional anticoagulant and antiplatelet agents to include a total of 16 studies and 11 comparators [10]. The previous NMAs of NOACs acknowledge the limitation of heterogeneity across the included trials and subgroup analyses have been performed to explore heterogeneity in two NMA publications [10, 14]. In Cameron et al., 2014, the results from subgroup analyses with respect to CHADS_2_ score, age and time to response differed marginally from those reported in the base-case analyses [10]. Similarly in Lip et al., 2016, some differences were observed in the results from the subgroup analyses with respect to CHADS_2_ score, secondary prevention and high quality anticoagulation with warfarin compared with those reported in the base-case [14]. None of the previous NMAs of NOACs have explored covariate effects using meta-regression methods.

### Exploring heterogeneity

In NMA trials must be sufficiently homogenous to be quantitatively combined, and all studies should be comparable in terms of potential effect modifiers across all interventions (or adjusted for using meta-regression). Furthermore, where NMA involves the combination of direct and indirect evidence these ‘two sources’ of evidence should be consistent [15]. The exploration of covariate effects is important in NMA because the presence of unaccounted treatment-covariate interactions can invalidate the assumptions which underlie NMA and bias results. In addition, it may be possible to identify treatment-covariate interactions which mean optimal treatment could differ for patients depending on their characteristics. Subgroup analysis and meta-regression are two approaches for exploring heterogeneity. Subgroup analyses splits the data into subgroups (i.e. categorical trial level covariate) and separate analyses are performed for each subgroup to allow for comparison of the estimates of treatment effect. Meta-regression methods allow for adjustments of differences on study-level characteristics by including a treatment effect interaction term. Where subgroup effects are suspected Dias et al., 2011, suggest that meta-regression is superior to running subgroup analyses [16]. Meta-regression allows a holistic analysis of exploring the impact of covariates on all of the data and allows the simultaneous consideration of multiple covariates. In addition meta-regression allows the incorporation of continuous covariates and formal testing of statistical significance of subgroups/continuous covariates.

### Baseline risk

Baseline risk is the underlying risk of the outcome of interest within a study population and represents a summary of both known and unknown risk factors. Baseline risk is a potentially important source of heterogeneity, particularly among studies where the baseline risk varies. The baseline risk is not a measurable quantity and clinicians only know about underlying risk through the patient’s characteristics which are measurable [17]. Baseline risk can also be seen as a proxy or surrogate for multiple (and potentially unmeasured) treatment confounding effects [17]. In this analysis baseline risk is defined as the event rate on placebo.

### Objectives

The objectives of this study were to i) perform an updated NMA in AF to include additional anticoagulant and antiplatelet comparators to warfarin and the NOACs; and ii) conduct a detailed exploratory regression analysis considering potentially important treatment modifying covariates including baseline risk. The decision question for this research is “what is the most effective treatment for stroke prevention in adults with AF” and if the answer to this question is the same for each patient or whether specific patient groups may differ in their response. The ‘decision set’ comprises all treatments that are eligible to be considered as the most efficacious, and in this analysis the decision set comprises adjusted dose VKA and the NOACs reflecting those treatments considered in clinical guidelines. The results of the analysis are presented for these comparators only. The rationale for including additional comparators outside of the decision set was to enable a more comprehensive network to allow for the exploration of covariate effects to include baseline risk.

## Methods

### Identification of studies

A number of meta-analyses based on systematic reviews comparing the relative efficacy and safety profiles of NOACs in AF have been published [10–14]. In many cases the evidence was restricted to Phase III RCTs for the established NOACs [11–14]. A targeted review of the literature for the most comprehensive evidence network for the numbers of studies and comparators identified a previous systematic review and NMA which included a single NOAC trial [18]. This NMA was based on previously identified data [18] and supplemented with new data from the three latest NOAC trials [6, 7, 9]. Potential effect modifiers extracted include baseline age, proportion of male patients, the proportion of patients with a history of stroke or transient ischemic attack (TIA), and mean baseline CHADS_2_ score.

### Statistical analysis

Ischaemic stroke was assessed because this outcome is the most frequent clinical manifestation of embolization associated with AF [19]. In addition this was the most commonly reported outcome across the trials in the updated systematic review. Study level covariates of interest explored in meta-regression analyses are the proportion of patients with a previous stroke/TIA, proportion of males, mean age, and the duration of study. There was insufficient data reported across the studies of the network to allow for the exploration of mean baseline CHADS_2_ score using meta-regression (5/23 studies reported mean baseline CHADS_2_ score).

The binary outcome data were modelled using a binomial likelihood and logit link [20]. All statistical models were fitted by adapting code written by the National Institute for Health and Care Excellence (NICE) decision support unit (DSU) for their evidence synthesis series [16, 20]. There are three potential general model specifications for including covariate interactions in an NMA framework [20, 21]. In this analysis a single covariate model was explored where a common study-level covariate effect versus the baseline treatment is assumed. This method implies that the level of covariate is assumed to modify the effect of each intervention in the same way relative to a common comparator. This approach was taken because the majority of direct comparisons within the evidence network are informed by single studies and insufficient data exists to fit more flexible covariate effects. The models are extended to adjust for potential effect modifiers by writing the study level effects as a linear function of the study level covariate. A meta-regression model on baseline risk (event rate in the placebo arm) using a standard model will ignore regression dilution bias and cause the covariate association to be overestimated [16, 22]. When including baseline risk as a covariate, the model is therefore modified to include the trial-specific baseline estimated by the model (rather than using that observed and ignoring uncertainty in its estimation) which is used as the covariate, taking into account the uncertainty in each baseline [16].

A Bayesian framework was used for modelling using Markov Chain Monte Carlo (MCMC) simulation, with the inclusion of vague prior distributions. Vague prior distributions for the study specific treatment effects and treatment effect sizes relative to treatment 1 (placebo) were in the form of a normal distribution with a mean of 1 and variance of 100^2^. The random effect (RE) models were run using a uniformly distributed prior distribution between 0 and 2 for the between study standard deviation (SD). All models were fitted using WinBUGS software (MRC Biostatistics Unit, Cambridge, UK). RE models were explored as it was deemed that heterogeneity was inevitable in the evidence network. Three chains were fitted with a burn in of 50,000 iterations to provide an effective means of checking for convergence for all models. Model convergence was assessed by analysing history and density plots, and Brooks-Gelman-Rubin (BGR) diagnostic plots [23, 24]. In addition, autocorrelation plots were assessed to detect the presence of auto-correlation in the chains [23, 24]. The inferences were made from data obtained by sampling for a further 50,000 iterations. It was not possible to conduct a formal assessment of consistency of the direct and indirect evidence as the evidence network only included loops of evidence from multi-arm studies and loops need to be formed using evidence from multiple sources to make this possible.

Model fit was measured by assessment of the posterior residual deviance and between-study heterogeneity. Model comparisons were based on comparing model fit in addition to the deviance information criterion (DIC). Leverage plots were also created to explore model fit. Leverage was calculated as the contribution to the effective number of parameters for each data point (pDi) and plotted versus the deviance residual of each data point. The leverage plots included lines (parabolas of the form c = x^2^+y) which demonstrate thresholds of the contribution to the DIC [20, 23]. Data points with a contribution of greater than a c = 3 threshold line were investigated.

## Results

**Fig 1:**
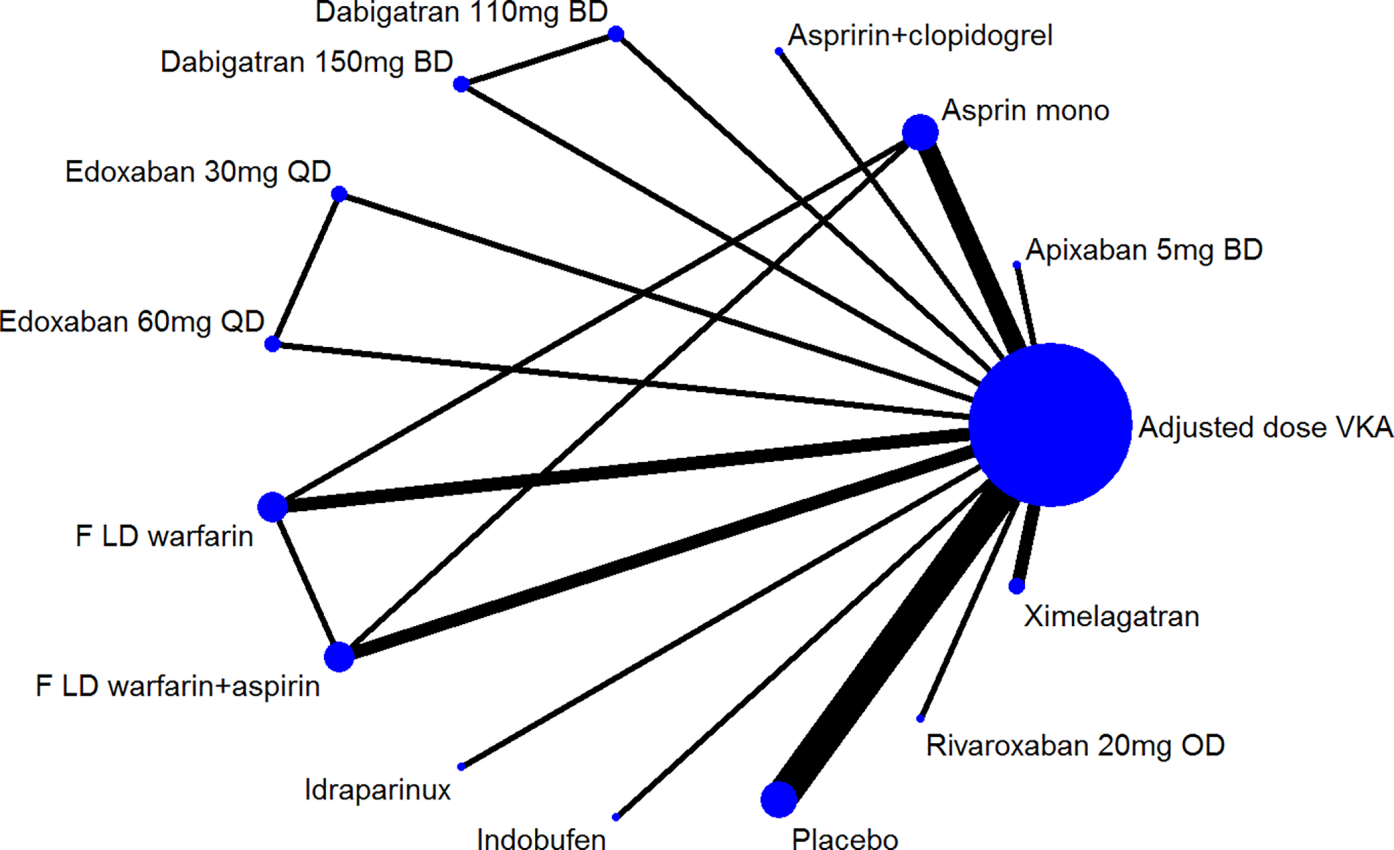
Evidence network for ischaemic stroke. The line thickness is proportional to the number of studies contributing to the treatment comparisons. Abbreviations: BD, twice daily dosing; FLD, fixed low dose; OD, once daily; QD, four times daily; VKA, vitamin K antagonist.

A total of 23 [6–9, 25–44] studies were identified for inclusion into the NMA, and of these 19 studies reported ischaemic stroke [6–9, 25–40]. The evidence network for ischaemic stroke is shown in Fig 1 and includes 15 comparators, representing the most comprehensive evidence network in AF published to date. All studies included an adjusted dose VKA treatment arm, and the loops of evidence are formed by multi-arm trials. The comparator set included fixed low dose warfarin with or without aspirin, aspirin monotherapy, aspirin plus clopidogrel, indobufen, idraparinux, triflusal and ximelagatran.

The trial design and baseline characteristics of the studies included in the ischaemic stroke evidence network are presented in Table A in S1 File. The study follow-up duration ranged from 11 months [32] to 42 months [37, 38] and the mean study follow-up was 22 months. The proportion of male patients ranged from 46% [35] to 100% [39] across studies and the mean proportion of male patients across all studies was 62%. The proportion of patients with a previous stroke or TIA ranged from 0% [36, 39] [34] to 55% [7] across studies, and the mean proportion of patients with a previous stroke or TIA across all studies was 18.5%. The mean age of patients ranged from 66 years [29] to 75 years [34] across studies and the mean age of patients across all studies was 72 years. The raw outcome data for ischaemic stroke are presented in Table B in S1 File.

### Unadjusted NMA model

**Fig 2:**
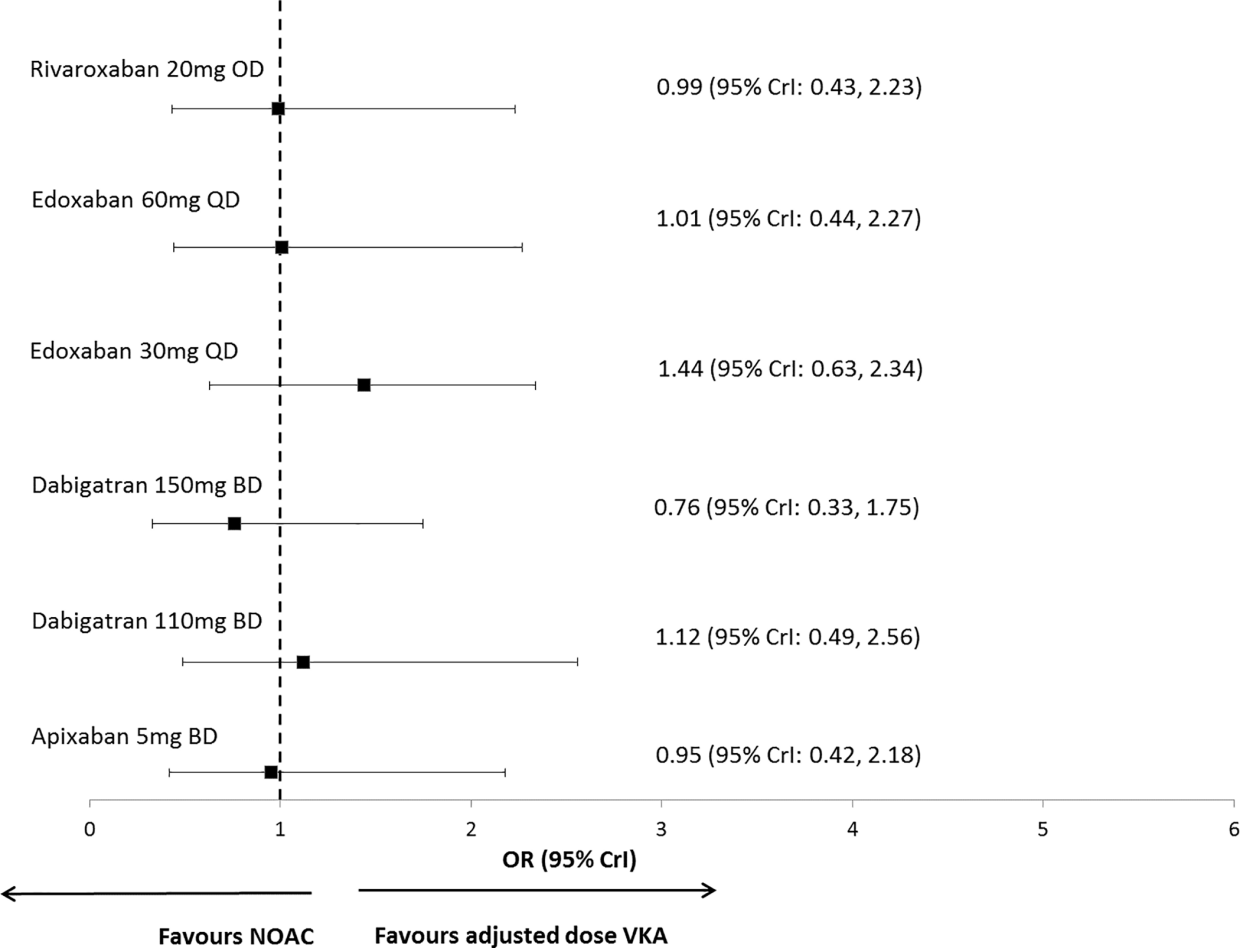
Forest plot of treatment effects of NOACs versus adjusted dose VKA from the RE unadjusted model. Abbreviations: BD, twice daily; FE, fixed effect; NOAC, novel oral anticoagulant; OD, once daily; QD, four times daily; VKA, vitamin K antagonist.

The results from the RE NMA model are provided in Table C in S1 File and are presented graphically in Fig 2. All 105 relative treatment effect results from this model are available from the authors upon request. The estimate of the between-study SD from this model was 0.26 (95% CrI: 0.02, 0.87) suggesting there is moderate heterogeneity in the evidence network. For the treatment comparison for which direct RCT data is available the point estimates from the NMA are consistent with the results from the direct RCT data, although they are associated with wider credible intervals reflecting the uncertainty in the estimate of between study heterogeneity in the model. The leverage plot (Figure A in S1 File) reveals a single observation with a larger magnitude of deviation and larger leverage than all other observations. This data point is due to the zero event observed in the adjusted dose VKA arm of the MWNAF trial which is an extreme and anomalous observation compared with the others studies [34].

### Binary model with adjustment for covariates

The following covariates were added individual to the RE binomial logistic model; i) proportion of patients with a previous stroke/TIA, ii) mean age, iii) proportion of males, and iv) follow-up time of study. Due to inconsistent reporting of the baseline characteristics two of the covariate adjusted models were reduced in size by one (age adjusted model) [28] or two studies (previous stroke/TIA adjusted model) [27, 28] compared with the base-case ischaemic stroke network of 19 studies [6–9, 25–40]. The binomial logit model fit statistics (DIC) were therefore compared with unadjusted models with the studies which did not report the relevant baseline characteristics removed to allow for an appropriate comparison of DIC. All covariate adjusted models contained the same number of comparators (n = 15) and the evidence network remained consistent with the network presented in Fig 1. The model fit statistics for the covariate adjusted models are presented in Table 1. In the covariate adjusted models the interaction coefficient is common for all treatments versus placebo.

**Table 1:** Model fit statistics for the four covariate adjustment analyses.

Statistic	Covariate adjusted RE Binomial logit model			
	Previous stroke/TIA	Proportion males	Age	Follow-up
Interaction coefficient, median (95% CrI)	0.01 (–0.06, 0.08)	-0.01 (–0.05, 0.03)	0.03 (–0.35, 0.38)	-0.31 (–1.71, 0.98)
DIC difference between adjusted and unadjusted models of the same data	0.76	1.46	–1.59	–1.69
Residual deviance	38.42^†^	41.47^‡^	40.04^¶^	40.17^‡^
Between study SD	0.43 (0.03, 1.58)^††^	0.29 (0.02, 1.04)^‡‡^	0.35 (0.02, 1.27)^¶¶^	0.30 (0.02, 0.98) ^‡‡^

Abbreviations: CrI, credible interval; DIC, deviance information criterion; RE, random effect; SD, standard deviation; TIA, transient ischaemic attack.

†compared with 38 data points

‡compared with 42 data points

¶compared with 40 data points

††compared with a SD of 0.34 (0.03, 1.26) in the unadjusted model

‡‡ compared with a SD of 0.36 (0.02, 0.87) in the unadjusted model

¶¶ compared with a SD of 0.31 (0.02, 1.06) in the unadjusted model.

None of the covariate adjusted models offered notable improvement in DIC compared with the unadjusted models. The 95% CrI of the interaction-coefficients crossed the null value in each adjusted model. Additionally, the between study SD of the RE adjusted models were either increased or marginally decreased compared with the respective unadjusted models.

### Binary model with adjustment for baseline risk

The baseline odds of ischaemic stroke in the placebo arms of studies in the evidence network ranged from 0.05 [40] [30][30][30][34] [34] [34]to 0.22 [30]. The mean baseline odds of an ischaemic stroke across all placebo arms was 0.10. A graphical representation of the odds of ischaemic stroke across the adjusted dose VKA arms of all studies is provided in Figure B in S1 File.

The model fit statistics from the baseline risk adjusted models and unadjusted models are provided in Table C in S1 File.

Relative treatment effect results for the head to head comparison of NOACs would remain constant across all baseline risks as the interaction regression terms cancel out within the model. The RE baseline risk model does not offer any improvement in DIC compared with the unadjusted model. Furthermore, the between-study SD is increased marginally in this model compared with the base-case RE model. The 95% CrI of the interaction coefficient does cross the null value implying there is little evidence to support the suggestion that the relative effectiveness of treatments is associated with the underlying risk of the event in the population.

In the baseline risk adjusted model a common covariate effect is assumed; related to a common comparator, placebo, noting that the coefficients are consistent for the treatment comparisons. When considering the leverage plots (Figure A in S1 File) the baseline risk model demonstrates an improvement in model fit compared with the unadjusted model. Notably, the extreme data point pulled right on the leverage plots of the unadjusted models is no longer an extreme data point and lies within the threshold lines.

## Discussion

The current NMA aimed to determine the relative efficacy of NOACs compared with warfarin in the prevention of ischaemic stroke while exploring and accounting for heterogeneity across the studies. The results of the unadjusted NMA are consistent with respect to point estimates and direction of treatment effects with previous analyses restricted to networks of NOAC trials only [14]. This is not unexpected as the evidence network for ischemic stroke in the current analysis does not include loops of evidence other than those from multi-arm trials and therefore no other studies in the current network are contributing to the unadjusted indirect comparisons of the NOACs. However, previous NMA analyses were performed using a fixed effect (FE) model which imply that no heterogeneity exists within the evidence. The results from previous analyses are associated with narrower 95% CrIs compared with this analysis. In this analysis all results are associated with 95% CrIs which include the null value as the RE model accounts for the uncertainty around the estimate of between study SD.

The exploration of heterogeneity using meta-regression methods suggested that age, previous stroke/TIA, gender and study follow-up did not affect the treatment effects relative to placebo. This is of particular interest for the ‘follow-up’ as a covariate because it provides evidence to support the assumption that study follow-up is unimportant for the analysis of ischaemic stroke despite variation in study follow-up across the trials. The CHADS_2_ score was developed to accurately predict the risk of stroke in patients with AF and was published in 2001 [45]. Given that many of the trials included within the evidence network pre-date the use of the CHADS_2_ score there was insufficient data reported across the studies to allow for the exploration of mean baseline CHADS_2_ score in a meta-regression. However, two previous NMAs in CHADS_2_ subgroups reported some differences compared to the base-case analyses [10, 14].

The baseline risk adjusted model suggested that the relative effectiveness of treatments (versus placebo) is not associated with the underlying risk of the event in the population. In the regression analyses a common covariate effect is assumed, related to a common comparator, noting that the coefficients are consistent for the treatment comparisons. While there are a range of treatments in the network the assumption of the common covariate effect seems sensible and potentially clinically plausible as all treatments are anticoagulants. However, it would be desirable to have further data to enable the exploration of alternative approaches of independent and exchangeable interaction coefficients. Baseline risk may be a proxy for unmeasured patient-level characteristics that influence a patient’s response to treatment [46]. It would be desirable to obtain IPD to facilitate an effort to relate treatment effects to IPD covariates in an attempt to investigate heterogeneity and identify particular characteristics which influence baseline risk and help make the results more applicable to patients facilitating more tailor-made patient decisions [17]. Unless IPD is available, using all trials in an area with NMA methodology cannot by itself identify interactions with treatment effect reliably and potentially prohibits data collected being used to its full advantage.

Many of the most recently published NMAs are restricted to adjusted dose VKA and NOAC treatments only. This analysis was more comprehensive and included additional anticoagulants and antiplatelets to the NOACs. This enabled a network which was sufficient (in terms of data points) to apply the exploration of covariate adjusted models. The inclusion of covariates in NMA are important to explain heterogeneity and inconsistency in analyses which will reduce bias in the analysis compared to when treatment effect modifiers (unbalanced across treatments) are not included. In addition, the inclusion of covariates in NMA has implications for precision medicine as it allow estimates of relative treatment effect beyond an overall mean, facilitating more tailor-made patient decisions.

Given the geometry of the evidence network none of the NOAC trials contributed to ‘loops of evidence’ and therefore, theoretically, the analysis could have been restricted to a star shape network including the NOAC trials only. However, a network of the four NOAC trials is insufficient in size to explore covariate or baseline risk effects as it would not include a relevant placebo arm for modelling baseline risk. It is also acknowledged that the approach taken in this analysis to include a more comprehensive evidence network does potentially increase heterogeneity within the network by additional unknown effect modifiers. We believe this to be the first analysis to explore the impact of baseline risk on a clinically important efficacy outcome in this indication. More generally, and regardless of indication, it is uncommon to find the exploration of baseline risk within NMA publications.

This study could be considered a ‘case study’ as the approach has potentially wide applications within any indication as to highlight the potential benefit of extending decision problems to include additional comparators outside of those of primary interest to allow for the exploration of heterogeneity. The extension of decision problems to include additional comparators outside of the decision set, to allow for covariate exploration, needs to be considered alongside the potential introduction of additional heterogeneity. Network size with respect to the amount of indirect evidence is related to this point and also remains an unsolved issue in NMA. A recent publication reported the results of an NMA case study whereby an evidence network was extended three times and concluded that the overall results were consistent across all networks and it did not increase heterogeneity or inconsistency [47]. There is therefore an unmet practical need to know how far to extend a network in the search for indirect evidence in health technology assessment.

## Supporting Information

S1 File Supporting Information. (DOCX)
