# Supplementary material for: Exploratory Network Meta Regression Analysis of Stroke Prevention in Atrial Fibrillation Fails to Identify Any Interactions with Treatment Effect
Source: PLoS One. 2016 Aug 25;11(8):e0161864. doi: 10.1371/journal.pone.0161864 (PMC4999289; doi:10.1371/journal.pone.0161864)
Supplement: S1 File — (DOCX) [file pone.0161864.s001.docx]

**S1 File. Supporting information**

**Table A: Study details and baseline characteristics for all identified for inclusion**

| Study | Treatment | Patients with prior stroke, % | Mean age, years | Proportion males, % | Mean CHADS_2_ score (SD) | ITT | Follow-up, months |
| --- | --- | --- | --- | --- | --- | --- | --- |
| CAFA (40) | AJD VKA (2.0-3.0) | 4 | 68 | 73 | NR | 187 | 15.2 |
| CAFA | Placebo | 3 | 67 | 76 | NR | 181 | 15.2 |
| RE-LY (8) | Dabigatran 110 mg BD | 20 | 71 | 64 | 2.10 (1.10) | 6015 | 24.0 |
| RE-LY | Dabigatran 150 mg BD | 20 | 72 | 63 | 2.20 (1.20) | 6076 | 24.0 |
| RE-LY | AJD VKA (2.0-3.0) | 20 | 72 | 63 | 2.10 (1.10) | 6022 | 24.0 |
| SPINAF (39) | AJD VKA (1.4-2.8) | 0 | 67 | 100 | NR | 260 | 21.6 |
| SPINAF | Placebo | 0 | 67 | 100 | NR | 265 | 20.4 |
| AFASAK 2 (37, 38) | Aspirin mono | 5 | 73 | 57 | NR | 169 | 42.0 |
| AFASAK 2 | AJD VKA (2.0-3.0) | 5 | 73 | 59 | NR | 170 | 42.0 |
| AFASAK 2 | F LD warfarin | 4 | 74 | 65 | NR | 167 | 42.0 |
| AFASAK 2 | F LD warfarin+aspirin | 9 | 73 | 59 | NR | 171 | 42.0 |
| PATAF (36) | Aspirin mono | 9 | 70 | 44 | NR | 141 | 33.4 |
| PATAF | AJD VKA (2.5-3.5) | 0 | 71 | 57 | NR | 131 | 36.7 |
| BAFTA (42) | Aspirin mono | 12 | 82 | 55 | NR | 485 | 32.4 |
| BAFTA | Adjusted dose VKA | 13 | 82 | 54 | NR | 488 | 32.4 |
| SIFA (35) | AJD VKA (2.0-3.5) | 49 | 72 | 49 | NR | 454 | 12.0 |
| SIFA | Indobufen | 50 | 73 | 46 | NR | 462 | 12.0 |
| MWNAF (34) | AJD VKA (2.0-3.0) | 0 | 74 | 75 | NR | 153 | 14.3 |
| MWNAF | F LD warfarin | 0 | 75 | 62 | NR | 150 | 14.6 |
| NASPEAF (41) | AJD VKA (2.0-3.0) | NR | 67 | 31 | NR | 232 | 28.8 |
| NASPEAF | Trifusal | NR | 67 | 29 | NR | 235 | 29.4 |
| ACTIVE W (33) | Aspirin+clopidogrel | 15 | 70 | 67 | 2.10 (1.10) | 3335 | 15.4 |
| ACTIVE W | AJD VKA (2.0-3.0) | 15 | 70 | 66 | 2.10 (1.10) | 3371 | 15.4 |
| AMADEUS (32) | AJD VKA (2.0-3.0) | 25 | 70 | 65 | NR | 2107 | 11.1 |
| AMADEUS | Idraparinux | 30 | 70 | 67 | NR | 1922 | 10.2 |
| BAATAF (31) | AJD VKA (1.5-2.7) | 3 | 69 | 75 | NR | 212 | 26.4 |
| BAATAF | Placebo | 3 | 68 | 70 | NR | 208 | 26.4 |
| EAFT (30) | AJD VKA (2.5-4.0) | 27 | 71 | 55 | NR | 225 | 27.6 |
| EAFT | Placebo | 30 | 70 | 58 | NR | 214 | 27.6 |
| SPAF (29) | AJD VKA (2.0-4.5) | 8 | 65 | 74 | NR | 210 | 15.6 |
| SPAF | Placebo | 8 | 66 | 70 | NR | 211 | 15.6 |
| SPAF II (28) | Aspirin mono | NR | NR | 76 | NR | 545 | 27.6 |
| SPAF II | AJD VKA (2.0-4.5) | NR | NR | 59 | NR | 555 | 27.6 |
| SPAF III (27) | AJD VKA (2.0-3.0) | NR | 71 | 59 | NR | 523 | 13.2 |
| SPAF III | F LD warfarin+aspirin | NR | 72 | 62 | NR | 521 | 13.2 |
| SPORTIF III (26) | AJD VKA (2.0-3.0) | 24 | 70 | 70 | NR | 1703 | 17.4 |
| SPORTIF III | Ximelagatran | 24 | 70 | 68 | NR | 1704 | 17.4 |
| SPORTIF V (25) | AJD VKA (2.0-3.0) | 18 | 72 | 69 | NR | 1962 | 20.0 |
| SPORTIF V | Ximelagatran | 19 | 72 | 70 | NR | 1960 | 20.0 |
| ROCKET-AF (7) | Rivaroxaban 20 mg OD | 55 | 71^†^ | 60 | 3.48 (0.94) | 7081 | 22.8 |
| ROCKET-AF | AJD VKA (2.0-3.0) | 55 | 71^†^ | 60 | 3.46 (0.95) | 7090 | 22.8 |
| ARISTOTLE (6) | Apixaban 5 mg BD | 19 | 70 | 65 | 2.10 (1.10) | 9120 | 21.6 |
| ARISTOTLE | AJD VKA (2.0-3.0) | 20 | 70 | 65 | 2.10 (1.10) | 9081 | 21.6 |
| ENGAGE-AF TIMI 48 (9) | Edoxaban 30 mg QD | 29 | 72^†^ | 61 | 2.80 (1.10) | 7034 | 33.6 |
| ENGAGE-AF TIMI 48 | Edoxaban 60 mg QD | 28 | 71^†^ | 62 | 2.80 (1.10) | 7035 | 33.6 |
| ENGAGE-AF TIMI 48 | AJD VKA (2.0-3.0) | 28 | 72† | 63 | 2.80 (1.10) | 7036 | 33.6 |
| WASPO (43) | Aspirin mono | NR | 83^†^ | 54 | NR | 39 | 12.0 |
| WASPO | AJD VKA (2.0-3.0) | NR | 84^†^ | 39 | NR | 36 | 12.0 |
| AFASAK (44) | Aspirin mono | 4 | 75 | 55 | NR | 336 | 24.0 |
| AFASAK | AJD VKA (2.8-4.2) | 5 | 73 | 53 | NR | 335 | 24.0 |
| AFASAK | Placebo | 4 | 75 | 54 | NR | 336 | 24.0 |

Abbreviations: AJD, adjusted dose; BD, twice daily; CHADS_2_, congestive heart failure, hypertension, age =75 years, diabetes mellitus, stroke; F LD, fixed low dose; ITT, intention to treat; NR, not reported; OD, once daily; QD, four times daily; SD, standard deviation; VKA, vitamin K antagonist.
†Median

**Table B: Data available for ischaemic stroke. Bold ORs indicate statistically significant results**

| **Study** | **Treatment** | **ITT** | **Number of events** | **Study level OR (95% CI)** |
| --- | --- | --- | --- | --- |
| CAFA (40) | AJD VKA (2.0-3.0) | 187 | 5 | 0.53 (0.17, 1.60) |
| CAFA | Placebo | 181 | 9 | control |
| RE-LY (8) | Dabigatran 110mg BD | 6015 | 159 | 1.12 (0.89, 1.40) |
| RE-LY | Dabigatran 150mg BD | 6076 | 111 | **0.77 (0.60, 0.98)** |
| RE-LY | AJD VKA (2.0-3.0) | 6022 | 143 | control |
| SPINAF (39) | AJD VKA (1.4-2.8) | 260 | 4 | **0.20 (0.07, 0.60)** |
| SPINAF | Placebo | 265 | 19 | control |
| AFASAK 2 (37, 38) | Aspirin mono | 169 | 5 | 0.62 (0.20, 1.94) |
| AFASAK 2 | AJD VKA (2.0-3.0) | 170 | 3 | 0.37 (0.10, 1.40) |
| AFASAK 2 | F LD warfarin | 167 | 5 | 0.63 (0.20, 1.96) |
| AFASAK 2 | F LD warfarin+aspirin | 171 | 8 | control |
| PATAF (36) | Aspirin mono | 141 | 4 | **1.88 (0.34, 0.46)** |
| PATAF | AJD VKA (2.5-3.5) | 131 | 2 | control |
| SIFA (35) | AJD VKA (2.0-3.5) | 454 | 10 | 0.56 (0.25, 1.22) |
| SIFA | Indobufen | 462 | 18 | control |
| MWNAF (34) | AJD VKA (2.0-3.0) | 153 | 0^†^ | 0.09 (0.00, 1.59) |
| MWNAF | F LD warfarin | 152 | 5 | control |
| ACTIVE W (33) | Aspirin+clopidogrel | 3335 | 90 | 2.20 (1.52, 3.18) |
| ACTIVE W | AJD VKA (2.0-3.0) | 3371 | 42 | control |
| AMADEUS (32) | AJD VKA (2.0-3.0) | 2107 | 20 | 1.41 (0.70, 2.84) |
| AMADEUS | Idraparinux | 1922 | 13 | control |
| BAATAF (31) | AJD VKA (1.5-2.7) | 212 | 2 | **0.14 (0.03, 0.64)** |
| BAATAF | Placebo | 208 | 13 | control |
| EAFT (30) | AJD VKA (2.5-4.0) | 225 | 16 | **0.34 (0.19, 0.64)** |
| EAFT | Placebo | 214 | 39 | control |
| SPAF (29) | AJD VKA (2.0-4.5) | 210 | 6 | **0.34 (0.13, 0.87)** |
| SPAF | Placebo | 211 | 17 | control |
| SPAF II (28) | Aspirin mono | 545 | 37 | 1.48 (0.88, 2.48) |
| SPAF II | AJD VKA (2.0-4.5) | 555 | 26 | control |
| SPAF III (27) | AJD VKA (2.0-3.0) | 523 | 11 | **0.24 (0.12, 0.47)** |
| SPAF III | F LD warfarin+aspirin | 521 | 43 | control |
| SPORTIF III (26) | AJD VKA (2.0-3.0) | 1703 | 46 | 1.45 (0.92, 2.29) |
| SPORTIF III | Ximelagatran | 1704 | 32 | control |
| SPORTIF V (25) | AJD VKA (2.0-3.0) | 1962 | 36 | 0.8 (0.51, 1.24) |
| SPORTIF V | Ximelagatran | 1960 | 45 | control |
| ROCKET-AF (7) | Rivaroxaban 20mg OD | 7081 | 218 | 0.99 (0.82, 1.19) |
| ROCKET-AF | AJD VKA (2.0-3.0) | 7090 | 221 | control |
| ARISTOTLE (6) | Apixaban 5mg BD | 9120 | 149 | 0.96 (0.76, 1.20) |
| ARISTOTLE | AJD VKA (2.0-3.0) | 9081 | 155 | control |
| ENGAGE-AF TIMI 48 (9) | Edoxaban 30mg QD | 7034 | 333 | **1.44 (1.21, 1.71)** |
| ENGAGE-AF TIMI 48 | Edoxaban 60mg QD | 7035 | 236 | 1.00 (0.84, 1.21) |
| ENGAGE-AF TIMI 48 | AJD VKA (2.0-3.0) | 7036 | 235 | control |

ADJ, adjusted dose; BD, twice daily; CI, confidence interval; F LD, fixed low dose; ITT, intention to treat; OD, once daily; OR, odds ratio; QD, four times daily; VKA, vitamin K antagonist

† Continuity correction applied to calculate study level OR as outlined within the Cochrane handbook

**Table C: Model fit statistics for baseline risk models compared with the base-case models for ischaemic stroke. A bold result indicates 95% CrI does not cross the null value**

| Model fit statistic | RE base-case | Adjusted for baseline risk RE |
| --- | --- | --- |
| DIC | 283.50 | 284.71 |
| Residual deviance (mean) [compared with 42 data points] | 41.89 | 40.11 |
| SD, median (95% CrI) | 0.26 (0.02, 0.87) | 0.27 (0.02, 0.88) |
| Interaction coefficient (95% CrI) |  | 0.39 (-0.46, 1.04) |

Abbreviations: CrI, credible interval; DIC, deviance information criterion; RE, random effect; SD, standard deviation.

**Figure A: Leverage plots**

1: Leverage plot for unadjusted RE Binomial logit model
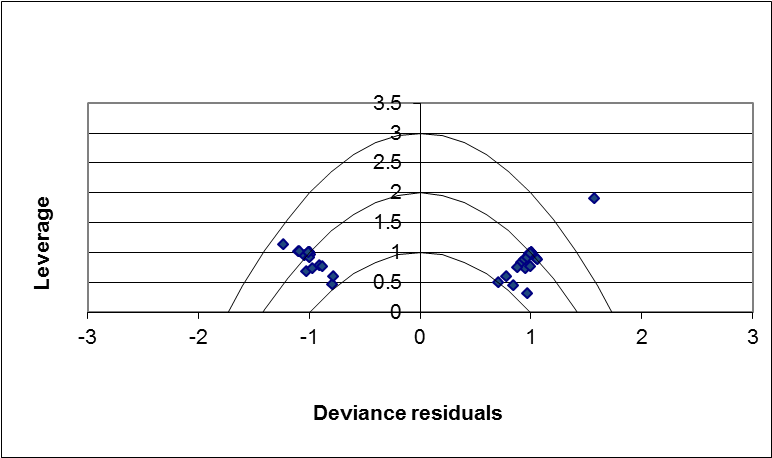


2: Leverage plot for RE baseline risk adjusted model (placebo reference)


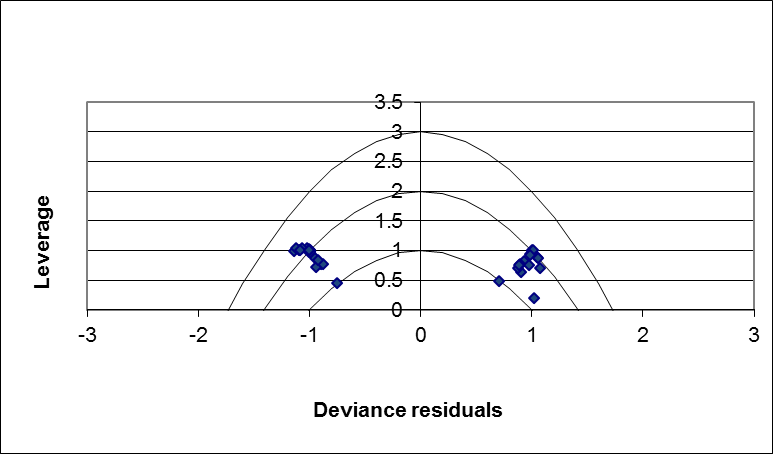


Abbreviations: RE, random effect.

**Figure B: Bar chart of mean odds of an ischaemic stroke in placebo arm of each study in the evidence network (n=5)**
